# Address of the Retiring President of the Fulton County Medical Society

**Published:** 1915-01

**Authors:** Stephen T. Barnett

**Affiliations:** Candler Building, Atlanta, Ga.


### Journal-Record of Medicine

Successor to Atlanta Medical and Surgical Journal, Established 1855
and Southern Medical Record, Established 1870

**OWNED BY THE ATLANTA MEDICAL JOURNAL COMPANY**

PUBLISHED MONTHLY

*Official Organ Fulton County Medical Society, State Examining Board, Presbyterian Hospital, Atlanta, Birmingham and Atlantic Railroad Surgeons' Association, Chattahoochee Valley Medical and Surgical Association, Etc.*

EDGAR BALLENGER, M. D., Editor
BERNARD WOLFF, M. D., Supervising Editor
A. W. STIRLING, M. D., C. M., D. P. H.; J. S. HURT, B. Ph.. M.D.
GEO. M. NILES, M. D., W. J. LOVE, M. D., (Ala.); Associate Editors
R. R. DALY, M. D., Associate Editor
E. W. ALLEN, Business Manager

**COLLABORATORS**

W. F. WESTMORELAND, M. D., General Surgery
F. W. McRAE, M. D., Abdominal Surgery
H. F. HARRIS, M. D., Pathology and Bacteriology
E. B. BLOCK, M. D., Diseases of the Nervous System
MICHAEL HOKE, M. D., Orthopedic Surgery
CYRUS W. STRICKLER, M. D., Legal Medicine and Medical Legislation
E. C. DAVIS, A. B., M. D., Obstetrics
E. G. JONES, A. B., M. D., Gynecology
R. T. DORSEY, JR., B. S., M. D., Medicine
L. M. GAINES, A. B., M. D., Internal Medicine
GEO. C. MIZELL, M. D., Diseases of the Stomach and Intestines
L. B. CLARKE, M. D., Pediatrics
EDGAR PAULLIN, M. D., Opsonic Medicine
THEODORE TOEPEL, M. D., Mechano Therapy
A. W. STIRLING, M. D., Etc., Diseases of the Eye, Ear, Nose and Throat
BERNARD WOLFF, M. D., Diseases of the Skin
E. G. BALLENGER, M. D., Diseases of the Genito-Urinary Organs

VOL. LXI. ATLANTA, GA., JANUARY, 1915. NO. 10

### ADDRESS OF THE RETIRING PRESIDENT OF THE FULTON COUNTY MEDICAL SOCIETY.

STEPHEN T. BARNETT, M. D.

Gentlemen of the Fulton County Medical Society:

It is one year ago tonight since you honored me by electing me by your suffrage to the highest gift in your power.

Most thoroughly have I felt the distinction bestowed. You have made the duties of your presiding officer a pleasure by your unfailing courtesy, not only during ordinary meetings and discussions, but also upon the few occasions when we have joined in debate upon differences of opinions.

The papers which have been presented during the year have been well prepared, as a rule, and those who have failed to read after having had notices sent out, have been remarkably few in number. The ones put down for discussion, on the contrary, have been very derelict in appearing. This has not always, by any means, been their fault, but the blame should be assumed by your president. For I believe those who are assigned to lead in the debate should be given longer notice of what is expected of them and that the essayist should give either to the President or Secretary a brief outline of how he will treat his subject, thus allowing the leaders of the discussion time to look into the matter which will be presented. This will or should give us five minutes of digested thought instead of a rambling discourse upon subjects oftentimes entirely foreign to the title.

Another criticism which I believe is merited and which I know arises from an innate disposition not to be disagreeable, is that there is too much of a tendency upon the part of those discussing papers to speak only of the laudatory things. Honest criticism should be welcomed by us all, for by such frank, though courteous, expression of our difference of opinion with the writer the members are instructed and he himself is a gainer. We are all prone to fall into ruts. We study a given problem very often under the same light and at the same angle, forgetful of the fact that there may be other viewpoints. So engrossed do we become with our presumably proven premises that, even admitted that our deductions are faultless, we lose sight of the fact that our assumed axioms may indeed be nothing but theories. One of our confreres, possibly no better versed in the subject than ourselves, opens our eyes, because he views it from a different standpoint. Nor should we take offense at honest criticisms. For the primary object of our meetings is to learn. Neither should we ever lose sight of this fact: Whatever extraneous matter may be brought into our debates, the cardinal principle of our organization is our general uplift and protection. How this may be best obtained will always be open to discussion and possibly even heated debates.

After a year of watching the proceedings upon the floor from the vantage point of the presiding chair, I believe it may not be amiss to speak of what might be with profit changed in our programs and possibly even in our By-Laws. For many years we have stuck religiously to papers which have oftentimes been but resumes of what some text-book may have elucidated without not only an original idea, but not even illustrated by a case in point. Text-books are often out of date when printed. How then can we benefit through such means? Or is there some remedy to apply to the deficiency? I think there is. The question is, how can we do it?

Part of it I believe could be most easily accomplished, though possibly at some individual inconvenience. Other parts will be most difficult. First, let us have our essays, but try to insist upon the writer giving the matter, not only the latest which the text-books may have, but also what the last periodicals have to say, supplemented by his own personal experience. Will it be possible to carry out this insistence? Yes, if we have the hearty co-operation of those who will agree to discuss. For if they have had time sufficient to get up on these questions, to read the latest Journals, the writer will soon be forced to do so himself.

Second, let us have symposiums more frequently, running over more than one meeting, if needs be, in which either by assignment or preference various aspects of the problem may be gone into. In this manner the question in its entirety will be brought before us, with possibly one or more illustrative cases. Let your committee limit the writers to specified times, from which there should be no appeal. In this way the discussion will not drag interminably until we all want to get home.

Third, a larger number of reviews of the latest advances in the different branches, as advocated at our last meeting by Dr. Roy. This will keep us all abreast of what is happening of interest in the various fields of medical endeavor without wading through a mass of material which may be of no moment to us individually. A committee of one from each branch could undertake to keep us informed, though not necessarily doing tho work themselves.

Fourth. This I find to be the hardest to solve. In looking back over the minutes of preceding years I find it from time to time under discussion, even as far back as 1894. At one time through the energy and money of one of our members it seemed likely to be successfully kept up. I refer to a working library. It is not necessary to go into details of its advantages which are patent to all. Suffice to say, that it cannot be expected, except in a few instances that we can all buy all the magazines which are necessary to keep us abreast of medical progress and yet some of what I have said before is dependent upon our free access to these aids. We have 250 members. Surely with that number with steadfast endeavor and possibly sacrifice on the part of some we could obtain what we all would like to see accomplished.

I hope your incoming executive will show more resource than I have in this matter particularly, as well as in others. In this connection and almost a corollary of it, I would like to call your attention to our cramped quarters. Fourteen years ago we were wont to meet with eight or ten members in a small room in what is now the Empire Life Building. Later we moved for a year or two into the Kimball with hardly any increase in our attendance. At that time through the courtesy of the Library officers we were extended the privilege of the whole end of this basement. In a few years a section was cut off and now recently the space assigned to us has been further reduced and this despite the fact that our membership has increased from about 100 to its present enrollment of 265 and at the same time our average attendance has gone up from about 8 to over 50. So I feel, as has been expressed by Dr. Daly in a motion at a former meeting, that we are gradually being crowded out. By common consent this was allowed to lay over for discussion at this meeting. I feel that this matter of a library and more commodious quarters should be under discussion at one and the same time, for in a vital way they are related one to the other and we should not do anything in regard to the room without having the other in mind as well. Many solutions of the problem have been offered, but so far, none that seems feasible. I trust that we may soon work it out.

Another means of advancing the interest in the Society and one which from time to time we have employed, is that of securing some authority of renown from another city who shall come and give us first hand the result of his labors, instruct and stimulate and inspire us to greater things ourselves. This has many advantages which can hardly be overestimated. Early in this year you will remember how much of profit as well as of pleasure was ours in listening to Dr. Henson, of Jacksonville. Two others had been arranged for, but unfortunately circumstances were such that we could not insist on their presence. If we could have two or three such lectures during each year we would gain much thereby.

Another question which has always been a bone of contention and possibly always will be, is that of discipline. I do not wish to open up closed things nor be thought to have any but the highest interest of the Society at heart, for I assure you that there is nothing personal in what I am about to say. It has been, however, brought to a focus quite recently by the action of a part of your Board of Censors in handing in their resignations. Whatever might have been the motives that prompted their action is beside the question. Whether they were right or wrong is immaterial. But what is of paramount interest to the Society and from which there is no escape, is that all bodies must have some prescribed method of examining into the fitness of applicants for membership and to pass upon infractions of the rules and laws which those bodies have chosen as their standards. Our individual method is through a Board of Censors whose election is laid down in our By-Laws. The duties are onerous and disagreeable. I, myself, have passed through the ordeal. They are very apt to be misjudged, to be misquoted and even maligned. Friendships have even been sacrificed, because of misunderstandings. Hence, it is a position not sought after and yet it should be filled by our very best and most level-headed men. They should have your support; make the position as easy as possible, and if compatible with your ideas of justice and moderation, follow their suggestions. If you differ, do so, of course, frankly, but at least do not misjudge their motives. In this connection I might say I have for some years felt that there should be a somewhat marked change in the procedure of our trials, which would mitigate the disagreeable task of this committee. I believe a committee should be appointed by your incoming President to draft certain changes which will work towards greater harmony, I believe, too, it would be a good precedent for this Society to adopt the idea which is prevalent in some secret orders of progression of their officers. For in this way you could reward these gentlemen who may have served as censor for the honorary and less abused places of Vice President and President.

I would like to call attention to the fact that we are primarily, of course, physicians, but we are also citizens of Atlanta. It is our duty, therefore, to further as far as possible everything that is for the civic good and betterment. There are many things that we have already done along that line. There are many others, though, which we could well back for the general upbuilding, some of which are not medical. Anything which benefits Atlanta benefits us individually and collectively. Because it may benefit one of us more than another is no reason to fight it. And herein we can be of enormous help not only to the citizens generally, but more particularly to ourselves. Boost, don't knock. And here, too, can we make our united help tell to the greatest advantage. Every man, woman or child who can be induced to make Atlanta their home is a potential help to us all. Every patient whom we may bring to Atlanta benefits, of course, the individual physician most, but he is indirectly a help to us all, physicians especially. For we are in that way educating our neighbors to look toward Atlanta when things medical are concerned. But when once turned this way they become also an asset either direct or indirect to every merchant and business man in the city. Co-operation should be our watchword.

Don't destroy simply because you cannot enjoy. Atlanta's success is your success. Atlanta's success has been builded by the success of the individual plus co-operation. So let us all pull together for the success of the city of Atlanta, for the success of her individual citizens, for the success of the medical fraternity as a whole and its upbuilding and particularly for the success of the Fulton County Medical Society, feeling well content if these others are advanced our own is assured.

Candler Building, Atlanta, Ga.

December 17, 1914.

